# The Effect of The Cognitive Behavioral Therapy and Pharmacotherapy on Infertility Stress: A Randomized Controlled Trial 

**Published:** 2013-09-18

**Authors:** Mahbobeh Faramarzi, Hajar Pasha, Seddigheh Esmailzadeh, Farzan Kheirkhah, Shima Heidary, Zohreh Afshar

**Affiliations:** 1 Fatemeh Alzahra Infertility and Reproductive Health Research Center, Babol University of Medical Sciences, Babol, Iran; 2 Department of Psychiatry, Faculty of Medicine, Babol University of Medical Sciences, Babol, Iran; 3 Department of Psychology, Payame Noor University, Tehran Branch, Iran; 4 Library of Center, Mazandaran University of Medical Sciences, Sari, Iran

**Keywords:** Infertility, Stress, Cognitive Behavior Therapy, Fluoxetine

## Abstract

**Background:**

Infertility has been described as creating a form of stress leading to a variety of psychological problems. Both psychotherapy and pharmacotherapy are effective treatments for infertility stress. The aim of this study was to evaluate the effectiveness of cognitive behavioral therapy along with fluoxetine for improvement infertility stress in infertile women.

**Materials and Methods:**

In a randomized controlled clinical trial, 89 infertile women with mild to moderate depression (Beck scores 10-47) were recruited into the following three groups: i. cognitive behavior therapy (CBT), ii. antidepressant therapy, and iii. control group. Twenty-nine participants in the CBT method received gradual relaxation training, restructuring, and eliminating of negative automatic thoughts and dysfunctional attitudes to infertility for 10 sessions. Thirty participants in the pharmacotherapy group took 20 mg fluoxetine daily for 90 days. Thirty individuals in control group did not receive any intervention. All participants completed fertility problem inventory (FPI) and the Beck Depression Inventory (BDI) at the beginning and end of the study. We applied Chi-square paired t test, ANOVA and Turkey’s test to analyze the data.

**Results:**

The mean of the infertility stress scores in CBT, fluoxetine, and control groups at the beginning and end of the study were as follows, respectively: 3.5 ± 0.62 vs.2.7 ± 0.62 (p<0.05), 3.5 ± 0.53 vs.3.2 ± 4.4 (p<0.05), and 3.4 ± 0.55 vs. 3.5 ± 0.48. In CBT group, the mean scores of social concern, sexual concern, marital concern, rejection of child-free lifestyle, and need for parenthood decreased meaningfully compared to those before starting the therapy. But in fluoxetine group, mean score of women sexual concern out of those five main problems of infertility reduced significantly. Also, fluoxetine and CBT reduced depression compared to the control group.

**Conclusion:**

CBT improved the social concerns, sexual concerns, marital concerns, rejection of child-free lifestyle, and need for parenthood more than floxitine group. Thus, CBT was not only a reliable alternative to pharmacotherapy, but also superior to fluoxetine in resolving and reducing of infertility stress (Registration Number: IRCT2012061710048N1).

## Introduction

The experience of infertility, defined by some as the infertility crisis, accompanies with physical, economical and social stress that affect all aspects of individual’s life. Maybe it can be said that infertility is one of the most stressful events in infertile people’s life (1, 2). Infertility and psychological difficulties are interrelated with each other. Some researchers believe the psychological difficulties as the cause of infertility, and call it, "stress hypothesis". They believe infertility as a psychosomatic problem. They also investigate the effect of psychological mood on neuroendocrine activities and the pregnancy rate. A study by Gallinelli et al. showed that there are correlations between stress, immunity, and fertility. Their result showed that there was higher serum cortisol and corticotrophin-releasing hormone (CRH) in infertile women than healthy control (3), while the similar studies suggest that higher level of stress accompanies with lower level of success in infertility therapy (4-7). Feeling of threat, sexual concern, guilt, hopelessness and marital problems are related to infertility (8).

But others, who have more supporters, know psychological stress as the result of infertility. According to this thesis, the experience of infertility affects the infertile couple with deep emotional tensions which is the fixed source of psychological and social dreads (9). Various studies reported a rise in anxiety and depression, and also decrease in self-confidence (10-15). The couples’ emotional reaction should be concerned from the very point of diagnosis of infertility, during therapy until success or failure of the process (16). At the moment, there is an international agreement that infertility centers should consider psychological problems. Kainz believes the role of a psychologist in infertility therapy process is investigation of cognitive documents, sexual problems, and marital relationships, while detecting and curing psychosomatic problems relating to fertility is possible through application of cognitive behavioral therapy (CBT) (17). Psychological council regarding the way of adaption with problem is a good way to avoid failure and hopelessness in infertile women (18). Terzioglu showed that infertile couples who received daily information and support during treatment have lower anxiety and depression scores, indicating higher life satisfaction than control groups (19). Similar studies showed that teaching confronting methods to infertile women gives rise to mental health and reduces depression (20). CBT is a psychotherapeutic approach based upon a combination of basic behavioral and cognitive research. The similar studies showed that psychotherapy is a reliable alternative to pharmacotherapy in order to decrease anxiety and to promote the mental health of infertile women (11, 21). Despite of widespread belief in the worthiness of CBT in the treatment of a variety of psychological problems, relatively, few studies have evaluated the effectiveness of psychosocial interventions in the field of infertility. There have been no published studies or randomized controlled prospective trials for adequate comparison of the impact of group psychological inventions with pharmacotherapy on improvement of infertility concerns. This study evaluated the different methods in order to achieve the best technique for improvement of infertility stress, while answering this question of whether CBT is a reliable alternative method to fluoxetine in this regard.

## Materials and Methods

A randomized controlled clinical trial was conducted in Fatemeh Alzahra Infertility and Reproductive Health Research Center of the Babol University of Medical Sciences, Babol, Iran, for nine months during 2007.

**Fig 1:**
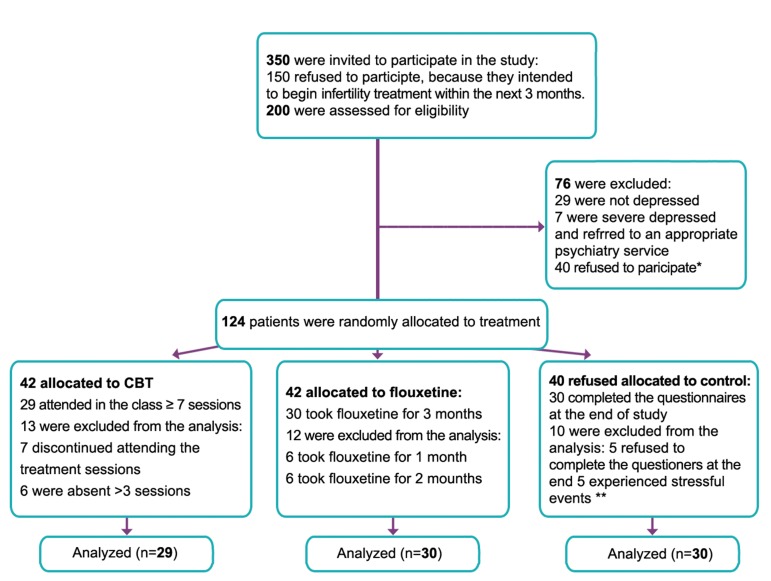
Flow diagram of participants through each stage of randomized controlled trial. *; Some of 40 depressed women refusing to participate in the study denied their mood disorders, while some of them believed that they could find relief from depression without medication intervention. Some of them were concerned if they began their treatment course, their family would mark them as mental patients. **; Stressful events, like family death, heavy accidents, pregnancy, etc, affected the stress infertility or depression tests, so these women were excluded from the analysis.

After coordination and receiving confirmation from Fatemeh Alzahra Infertility and Reproductive Health Research Center of Babol University of Medical Sciences (Iran), infertile women who had dossier the center, were recruited for this study. The participated women had the following characteristics: less than 45 years of age, more than five years of education, more than two years of infertility, having at least one *in vitro* fertilization (IVF), no fertility treatment for a three-month interval after IVF, no practicing in any relaxation techniques, no participating in any support group, no taking any psychotherapy, and no assisted reproductive therapy (ART). Five midwives at the center conducted telephone invitation with potential participants. Among 350 invitations, 200 patients accepted to enter the study, so after giving the informed consent form, they were referred to the center. Subsequent to completing the demographic questionnaire and the Beck Depression Inventory (BDI), a psychologist conducted a face-toface interview. Women either with a score ≤9 or >47 on the BDI, or clinical diagnosis of severe depression were excluded from the study. Thus, only women with minimal, mild, and moderate depression (Beck score 10-47) were included in the study. At the end of the interview session, participants were randomly selected for one of following three groups, CBT, fluoxetine and control, through a computer program. The block randomization was by a computer randomize list in which participants were labeled randomly to number 1-124 by an investigator with no clinical involvement in the trial, like: numbers 1, 4, 7,… for CBT; number 2, 5, 8,… for fluoxetine; and numbers 3, 6, 9,… for control group. Figure 1 shows the flow diagram of participants through each stage of randomized controlled trial. Finally, 89 participants were randomly divided among three groups as follows: CBT (n=29), fluoxetine (n=30), and controls (n=30).

Participants in the CBT group were engaged a two-hour group cognitive behavior therapy session for 10 weeks. Progressive muscle relaxation was added to the sessions 5-10. Groups consisted of 8-12 members, and the therapist, as an expert psychologist, was trained for the CBT program. Therapy was conducted at the Psychiatry Department of the Babol University of Medical Sciences, Babol, Iran. The first three sessions provided patients with a general orientation to cognitive therapy and the causes of infertility.

In the first three sessions, a gynecologist explained the cause of infertility for each woman. Cognitive therapies included that dysfunctional attitudes to social concerns, sexual concerns, marital concerns, rejection of childfree lifestyle, and need for parenthood more required challenged (22). In sessions 4-6, irrational beliefs about the infertility challenged. Finally, sessions 7-10 taught participants varying techniques for maintaining the change of their dysfunctional beliefs about infertility (23). In addition, session’s 5-10 progressive muscle relaxation of Jacobson was added to the cognitive therapy of Beck model (24-26).

The pharmacotherapy group took a capsule of fluoxetine (20 mg) daily for 90 days. After the interview, 30 capsules, prescribed by a psychiatrist, were provided by the midwives of the center, monthly. The control group didn’t take any drug or psychological intervention. They completed two questionnaires at the beginning of the study and three months after the interview. In CBT group, subjects had therapy interventions, including IVF, anti depression or psychological drug during three months; however, some individuals with stressful events such as family death were excluded from analysis.

All participants completed the Fertility Problem Inventory (FPI) and BDI at the beginning and end of the study. FPI used was a reliable instrument to assess the infertility stress. This instrument contains 46 items and 5 subscales that cover infertile couple’s stress such as social concern, sexual concern, marital relationships, rejection of child-free lifestyle, and need for parenthood. Participants indicated their degree of agreement with each item on a sixpoint Likert scale ranging from "strongly disagree" to "strongly agree". Newton et al. (27), Cousineau and Domar (1) reported the internal consistency reliability was 0.22-0.66, testretest reliability was 83%, and its correlation with Beck Inventory was 60%. Also, Persian version of FPI is reported with high reliability (α=0.91) (28). There was a meaningful positive correlation (p<0.01) between Beck Depression Inventory and infertility stress test (0.75) (29). In this sample, the internal consistency reliability of FPI was 0.8. Also, its correlation with BDI was 63%. The mean score of FPI for infertile women was obtained after calculating the scores in every subgroup. The least mean score based on Likret index was 0, "strongly disagree", and the highest one was 6, "strongly agree". All aspects of this protocol were approved by the Ethics Committee of the Babol University of Medical Sciences.

All analyses were performed using SPSS software. Paired t-tests were used to compare the mean scores of FPI and depression tests in each study group, separately, before and after interventions. Analysis of variance was performed to compare the mean scores of the tests in the three study groups at the beginning, and also, at the end of the study. If there was meaning difference among three groups, Posthoc (Turkey’s test) test was used to compare the mean differences. It should be noted that results of this articles has been a part of extend project implemented for Fatemeh Alzahra Fertility and Infertility Health Research Center of the Babol University of Medical Sciences (2007). Comparison pharmacotherapy and psychotherapy in improvement depression, anxiety, and general health reported in previous publications (11, 21). This article focuses on comparison of two types treatment in reducing of infertility problems in dimensions of the social concerns, sexual concerns, marital concerns, rejection of child-free lifestyle, and need for parenthood.

## Results

The findings of research showed that there were no statistically significant differences among the three groups in age, education level, and the duration of infertility. The demographic characteristics of the study sample are summarized in table 1.

**Table 1:** Characteristics of diluting media used for control and four treatment groups


Criteria	CBT^a^ mean (SD)	Fluoxetine mean (SD)	Control mean (SD)	F^b^	P value

**Age (Y)**	28.3 (3.8)	29.8 (5.3)	28.4 (5.3)	0.8	0.4
**Education (Y)**	9.2 (2.4)	9.4 (4.2)	9.8 (3.9)	0.4	0.2
**Duration of infertility (Y)**	5.4 (3.9)	6.3 (3.4)	5.7 (4.4)	0.3	0.6


a; Cognitive behavior therapy and b; ANOVA was performed to compare the mean scores of groups.

Evaluating the mean of FPI scores in infertile women showed that rejection of child-free lifestyle had the highest score in all three groups among five main fertility problems (social, sexual, marital concerns, rejection of child-free lifestyle, and need for parenthood). Although fluoxetine decreased the overall mean scores of FPI, it could only decrease the mean score of sexual concerns among five main fertility problems. Also, mean scores of all five main fertility problems decreased significantly in CBT group in comparison to before starting the therapy. After CBT sessions, overall mean scores of FPI decreased significantly in comparison to before starting the therapy. There were no significant differences in mean scours of control group (Table 2). The results had showed an improvement in depression level in CBT (79.3%) and fluoxetine (50%) groups (p<0.001) compared to previously reported analysis (11).

**Table 2:** The mean scores of five main fertility problems in three groups of infertile women at beginning and end of the study


Groups	CBT^a^ mean (SD)			Fluoxetine mean (SD)			Control mean (SD)		

**Scales**	**Before**	**After**	**P**	**Before**	**After**	**P**	**Before**	**After**	**P**
**Social concerns**	2.9 ± 0.72	*2.1 ± 0.76	0.0001	2.9 ± 0.59	2.8 ± 0.55	0.709	2.9 ± 0.78	3.1 ± 0.96	0.066
**Sexual concerns**	3 ± 0.71	*2.6 ± 0.68	0.007	3.1 ± 0.67	*2.7 ± 0.67	0.041	2.9 ± 0.75	2.9 ± 0.60	0.425
**Marital concerns**	4 ± 0.89	*3.1 ± 0.97	0.020	3.8 ± 0.89	3.5 ± 0.70	0.144	3.7 ± 0.75	3.8 ± 0.59	0.388
**Rejection of childfree lifestyle**	4.1 ± 0.91	*3.4 ± 0.94	0.002	4.3 ± 0.77	4.2 ± 0.79	0.432	4.3 ± 0.78	4.2 ± 0.66	0.688
**Need for parenthood**	3 ± 0.73	*2.3 ± 0.76	0.0001	3.2 ± 0.99	3.2 ± 0.72	0.925	3.1 ± 0.74	3.2 ± 0.74	0.580
**Total score**	3.5 ± 0.62	*2.7 ± 0.64	0.0001	3.5 ± 0.53	*3.2 ± 0.44	0.045	3.4 ± 0.55	3.5 ± 0.48	0.373


a; Cognitive behavior therapy and Paired t test was used to compare the mean scores, before and after inventions.

## Discussion

The results of this study showed that the infertile women’s mean scores for all infertility stress dimensions (social, sexual, marital concerns, rejection of child-free lifestyle, and need for parenthood) were very high. The highest scores were related to inability to accept the life style without a child and marital relationships, which is in concordance to the similar study (4). Basically, for a woman, the need to have child is a fundamental issue, and is considered to be the normal process of life. It is also the symbol of reaching evolution, adulthood and womanish personality (30-32). The role of being a mother is the most important role, and playing this role is suitable for women (33). In different cultures, becoming a parent is a turning point, and is also assumed to be an important occurrence in couple’s life (34). Inability to have a child is a stressful condition for infertile couple (1) because both man and woman are frustrated, damaged and under too much pressure. They may also pay less attention to each other’s needs, so that their relationship will be cold and damaged (35). Alizadeh et al. (28) declared, "Infertile women tolerate too much pressure and this pressure could affect the couple’s interpersonal relationships, and also, can disrupt their marital and sexual relationship which finally leads to a critic condition". They also suggested that CBT psychotherapy is significantly effective on infertility stress. Domar et al. (36) in a controlled clinical trial showed "the impact of group psychological intervention on distress in infertile women". 

It evaluated CBT group and supportive psychotherapy group with control group. The results showed that the different stressful subjects relating to psychological indexes of intervention group, especially the CBT group, decreased in comparison to that of control group (36). Cousineau and Domar (1) believed that psychological interventions, especially proper training for adapting different skills in order to assess and mange the stress, had beneficial effects on infertile women. In a similar study by Gharaie et al. (37) the CBT trainings decreased stress and anxiety of infertile women undergoing the infertility therapy. Wallace also showed that stress and anxiety of patient decreased after counseling supports (38), while women in the CBT sessions reached a better mood, and their stress and anxiety decreased compared to control group (36).

Our results revealed a significant decrease in all infertility stress dimensions (social, sexual, marital concerns, rejection of childfree lifestyle, and need for parenthood) in CBT group, whereas in fluoxetine group, there was only a significant decrease in sexual concern dimension. Faramarzi et al. (11) and Antonuccio et al. (39) reported that CBT is not only a reliable alternative to pharmacotherapy, but also superior to fluoxetine in the resolving or reducing of depression and anxiety of infertile women. The other investigations also showed that using CBT could decrease all physical and psychological symptoms such as anxiety, depression, frustration, insomnia, etc (40). In addition, they declared that high level of distress is observed in infertile women who had the least emotional supports from families and spouses (30). So, the infertility centers should provide different therapy programs through which infertile women receive proper support and necessary trainings in order to learn how to manage the stress (41-43).

Our finding confirmed the management of depression in the both fluoxetine and CBT groups that complete description was previously reported by Faramarzi et al. (11). Some similar studies know CBT as the effective therapy for depression (44). Doing psychological interventions through CBT and supportive counseling could not only improve the depression, but also increase reproduction fertility in CBT group by 55%, supportive group by 54%, and control group by 20% (35, 45).

 Studies by Thorn reported that infertility counseling decreases the emotional burden of infertility during treatment, while psychological counseling not only supplies the vital emotional support, but also diminishes the drop-out rate in infertility treatment (46, 47). Also, there are results showing that psychological programs decrease negative feelings through group interventions by training necessary skills in order to decrease the depression (48, 49). There were a number of limitations in the current study. The first limitation was that 40 mild to moderate depressed women did not agree to enter treatment protocol. The second limitation was the number of dropouts from experimental and control groups. Fortunately, demographic characteristics of women were the same before and after dropouts. We determined that the number of dropouts did not bias the data in favor of the interventions. Third limitation was cultured band difference in north of IRAN that was a variable response to CBT or compliance for treatment. Forth limitation referred to the nature of two types of treatment. CBT group received more treatment than drug group, so the obtained positive results may be due to more effective counseling programs rather than anything specific about CBT.

## Conclusion

CBT was effective on decreasing fertility stress in dimensions of the social concerns, sexual concerns, marital concerns, rejection of child-free lifestyle, and need for parenthood. Also, CBT was superior to fluoxetine for resolving infertility stress. Of course, more studies are needed to investigate the effect of CBT on rate of reproduction fertility in infertile women. This project proposed that it is necessary to investigate the effectiveness of routine CBT to prevent infertility stress in infertile couples.
